# The Relief Funds for the War

**Published:** 1899-12-16

**Authors:** 


### The Hospital

A JOURNAL OF

The Medical Sciences and Hospital Administration.

VOL. XXVII.—No. 690.] EDITED BY SIR HENRY BURDETT, K.C.B., AND SOLOMON C. SMITH, M.D., M.R.C.P. [DECEMBER 16, 1899.

### THE RELIEF FUNDS FOR THE WAR.

If anything could be more thoroughly characteristic of English people than the dignified and patient calmness with which the public awaits intelligence from South Africa, it would be the extraordinary liberality with which private donations have been poured in for the subsidiary purposes of affording aid to the sick, the wounded, and the disabled; or for maintaining the standard of comfort of the wives, the children, and the dependents of the soldiers who are engaged in the war. The Lord Mayor has lately announced his confident expectation that the contributions received at the Mansion House would shortly amount to half a million sterling; and, in addition to this great central fund, local ones are springing up in many parts of England, or are being brought into existence with special reference to particular classes of wants or to particular classes of claimants. So large a number of ladies are employing themselves in the household fabrication of "Tam o' Shanter" sleeping caps for the combatants that it has been necessary to coin a new participle, "tamming," as a convenient means of describing the occupation. But the eagerness to contribute, unfortunately, has not in every case been guided by the degree of forethought which is always necessary for the wise expenditure of money. The Lord Mayor, when the Mansion House Fund was established, plainly announced that the sum received might be assigned by the donors to one of four principal objects, the future support of widows and orphans by the Patriotic Fund, the care of the sick or wounded during the war, the eventual support of disabled men, or the affording of present help to the wives, children, or families of the soldiers who were at the front. He intimated at the same time that all monies which were not specifically assigned by the donors would be given for the first of these objects. Notwithstanding this plain notice, a vast number of contributors have failed to afford any clue to their wishes, with the result that the amount allotted to the Patriotic Fund appears even now to be sufficient for all the probable calls upon it. The Lord Mayor has therefore been compelled to reconsider the original arrangement, and to announce that for the future all sums sent without special instructions will be applied at his own discretion in the direction in which they seem most needed at the time. There can be no doubt that the change of plan will be heartily welcomed by the public, and that it is eminently wise and timely. The Lord Mayor is in a position to command at once the most accurate information and the most skilful and experienced help; and his action will unquestionably be conducive to the accom plishment of the largest possible amount of good for which the money at his disposal will suffice.

Almost more important than money, however, will be the element of personal help and supervision through which alone the money can be distributed wisely. There is scarcely a locality in England which does not contain the wives and children of men called up from the reserves, and there is nothing which will tend more powerfully to the comfort and satisfaction of the army, and to the maintenance of that cheerfulness which is a step on the road to victory, than the knowledge that those they leave behind are cared for with sympathy as well as with money. Every parish in the United Kingdom should have its lady visitor or visitors for soldiers' wives; and the functions of these visitors should not terminate with inquiries into financial circumstances, and into the necessity of supplementing earnings or allowances by pecuniary gifts, but should extend to an endeavour to assume the position of friends and counsellors, ready to give a helping hand in any or every emergency, and prompt with news of engagements in which the regiment has taken part, and with such personal information, either positive or negative, as the official lists will afford. A soldier's wife should be able to feel confident that, if her husband is killed or wounded, the intelligence will soon reach her, not only through official but also through kindly channels, and that she will not be left alone to bear her trouble as best she may. Many members of the medical profession have already placed their services at the disposal of local committees for attendance on the women and children in case of illness, and the example cannot be too widely followed. A time has come in which it may be possible to remove the last vestige of that prejudice against the army which came into existence during the Peninsular War, as a not unnatural result of the character of the classes from which it was at that time largely recruited, and to restore it to its natural and historical position as an integral and cherished element of a great and united Empire. The accomplishment of this most desirable end is now to a great extent within the power of the women of Great Britain; and it is to be brought about, not by the mere expenditure of pence or pounds, but by the active display of womanly sympathy, founded upon common anxieties and upon national sisterhood.

